# Misinformation making a disease outbreak worse: outcomes compared for influenza, monkeypox, and norovirus

**DOI:** 10.1177/0037549719885021

**Published:** 2020-04

**Authors:** Julii Brainard, Paul R Hunter

**Affiliations:** Norwich Medical School, University of East Anglia, UK

**Keywords:** Agent-based models, norovirus, influenza, monkeypox, fake news, social networks

## Abstract

Health misinformation can exacerbate infectious disease outbreaks. Especially pernicious advice could be classified as “fake news”: manufactured with no respect for accuracy and often integrated with emotive or conspiracy-framed narratives. We built an agent-based model that simulated separate but linked circulating contagious disease and sharing of health advice (classified as useful or harmful). Such advice has potential to influence human risk-taking behavior and therefore the risk of acquiring infection, especially as people are more likely in observed social networks to share bad advice. We test strategies proposed in the recent literature for countering misinformation. Reducing harmful advice from 50% to 40% of circulating information, or making at least 20% of the population unable to share or believe harmful advice, mitigated the influence of bad advice in the disease outbreak outcomes. How feasible it is to try to make people “immune” to misinformation or control spread of harmful advice should be explored.

## 1. Introduction

Previously we constructed an agent-based model (ABM)^1,2^ about what could happen during a norovirus outbreak exacerbated by circulating misinformation (which we call “bad advice”). Bad advice is relevant when it changes human behavior to be riskier (that is, a higher risk of getting disease). Examples of possible risky behavior can include not washing hands, sharing food with ill people, not disinfecting potentially contaminated surfaces or fomites, sneezing, coughing, or vomiting in places where there is a high risk of spread onto food or surfaces, lack of disinfection, and other unprotected physical contact with infectious persons or their bodily fluids.

Some people in disease outbreak situations take few precautions to avoid getting disease, even when awareness of the outbreak is widespread and many official sources are widely disseminating information about how to avoid illness. Compliance with quarantine protocols during a 2003 outbreak of severe acute respiratory syndrome (SARS)-coronavirus was found to be uneven. Compliance related to risk perception, which in turn related to how trustworthy and credible sources of health advice were perceived to be, as well as difficulties respondents had in accepting parts of the situation they could not be in control of.^3^ During a large and well publicized norovirus outbreak at a Canadian university, 25% of symptomatic students were observed to not avoid contacts, while 17% of the observed cohort did not comply with recommended handwashing practices.^4^ In a 2019 survey in the UK,^5^ 14% of 2000 surveyed parents reported sending a child to school with symptoms of contagious chickenpox, violating school policies and official advice to quarantine such children.

That misinformation can be linked to taking fewer measures to effectively prevent disease transmission is especially well documented with respect to Ebola viral disease (EVD). For instance, persons affected by the West Africa outbreak (2013–2016) who believed other types of misinformation about EVD (e.g., that it can be airborne or transmitted via mosquito bites) were more likely to report practicing unsafe burial practices.^6^ Low trust in institutions and more belief in EVD misinformation were associated with fewer preventive behaviors in the Congolese outbreak that started in 2018.^7^ Following ineffective advice or disbelieving official recommendations often led to pursuing other self-care actions first and thus delayed actions that could reduce transmission (such as formally getting tested for EVD).^8^ Most sources that have studied disease-precaution behavior, especially vaccine refusal (all diseases), note that cultural identity outside perceived mainstream society is strongly positively linked to the tendency to reject expectations of “good citizen behavior,”^9–12^ including disease avoidance actions recommended by public health authorities.

Our objective was to model possible interactions between misinformation spread and disease outcomes. The modeling was done within an agent-based modeling^13^ environment, using Netlogo software.^14^ Most model parameters were available from sources reporting actual human behavior, such as about the speed and frequency of social media and real life information sharing. An ABM environment was attractive because it afforded many opportunities to include complex aspects of behavior and response that might vary individually. We use the term *complex* to distinctively mean processes that are thought to be inherently unpredictable and have uncertain outcomes (as opposed to the often misused near synonym *complicated*, which can be better interpreted as describing events that can be modeled using consistent albeit possibly very multi-faceted decision trees).

Complexity in our models meant that, for instance, clusters of individuals with frequent contact with each other (physical or information sharing contact) might have very different traits from the population averages. However, these differences could not be consistently predicted; nor could location or encounters with other agents. Location and chances of getting disease or encountering misinformation were best estimated for our purposes using reiterative modeling and probabilistic distribution of relevant attributes. These skewed traits could lead to localized hotspots or low activity zones of disease and/or information transmission. Multiple types of feedback loops could be in operation that affected behavior choices. These feedback processes could be modeled at individual level using an ABM. At the same time, many aspects of behavior and response seemed likely to be probabilistic but perhaps on uncertain distributions or with unclear central tendency points. The iterative nature of ABMs meant that we could not only try to use real world data to describe some behavior aspects in the models, but also use iterations to estimate what some central values for other behavior tendencies might be.

In the ABM that we constructed, disease spread was via direct or indirect physical contact, while misinformation spread was via social contact, particularly within social groups (“bubbles”). These social groups were constructed to have relatively similar susceptibility for believing the misinformation. Members of one’s bubble were often the same persons that were physically encountered, so these could be the same people with whom it was likely that the disease could be exchanged. Our initial work focused on norovirus because, although very common, gastrointestinal illness is rarely modeled in individual infectious disease models.^15^ Norovirus also had the advantage of being unlikely to cause flight or death (so those things could be defensibly not included in the model). The incubation period is relatively short so travel outside the residence area was excluded.

The purpose of this study is to adapt our misinformation (agent-based) model to other communicable diseases and outbreak conditions. We compare results for norovirus, influenza, and monkeypox (*Orthopoxvirus*). The latter diseases can be substantially different from norovirus in many respects (such as the mean incubation period) and are often not previously vaccinated for. Influenza is an important communicable respiratory illness globally, while monkeypox is an emerging disease of high concern that has triggered biosecurity concerns.^16,17^

These pathogens gave good opportunities to show that our modeling approach could be adapted to multiple types of disease and transmission risks.

## 2. Methods

### 2.1. Overview

Table 1 shows model assumptions and targets, separated by disease. Model assumptions and design features, such as the rate of information injections, are described in greater detail elsewhere.^2^ The underlying Netlogo model code is available from the authors upon request. At least 100 iterations were run for the most likely candidate thresholds or parameters to verify their reliability. Extra simulations (above 100 minimum) were run until we found that additional model runs did not produce mean or median estimates that were any closer to the target conditions (i.e., a monotonic state in target outputs had been achieved).

**Table 1.** Model assumptions and targets (stage 1).

	Norovirus	Influenza	Monkeypox
**Model baseline target** ***r*0 or generations**	*r*0 = 1.9^63^	*r*0 = 1.47^64^	Insufficient data to estimate *r*0^65^ but assume 75% outbreaks have ≤4 generations of p2p transmission^66^
**Targets in stage 2** **(worse *r*0 or gens)**	*r*0 = 2.66	*r*0 = 2.06	Increase to 75% outbreaks have ≤7 generations
**% agents who start model ill (infectious)**	2%	2%	1%
**Incubation period?**	36 hours (mean)^67,68^	48 h (mean), range 1–4 days; SD = 0.5 d^69^	12 d (mean)^70^ (range 7–17 d)
**Viral shedding pre-illness?**	Assumed none	Yes: 1 day before illness starts	Assumed none
**Viral shedding post-illness?**	Mean 48 hours	Only if illness <6 days	Assumed none
**Infectious period while ill**	Entire duration of illness^71–73^	First 6 days (mean) after symptoms onset, or until no longer ill (if <6 days)^69^	Entire duration of illness
**Duration of illness**	46 hours^71–73^	7 days, with SD = 1.75 day^69^	21 d (mean)^70^ (range 14–28 d)
**Chances of hospitalization**	0%	5%/d (when ill)	70%/d (only after 4 d)
**Case fatality rate**	0%^67^	0.65%^74^	Low because assumed high-income country setting, 1–2%^75^
**Vaccination available**	No	After 5 months^76^	After 18 wks^77^
**Vaccination efficacy**	Not applicable	100% if not incubating^78^	100% if <4 d after exposure, else 0^77^
**Vaccination uptake**	All those with TP >25th percentile of TP% & susceptible (or incubating <4 days if monkeypox)		
**Model tests relevant to disease transmission**	If neither side takes enough precautions AND viral shed risk is high enough		

CFR: case fatality rate; p2p: person to person; TP: take-precautions; gens: generations.

Notes: targets are from the literature (influenza pandemics after 2009). The CFR for monkeypox is plausible,^65^ but there is a lack of data from high-income country settings. The model assumes no shortage of vaccines (once available, there is always enough to meet demand). Delay in influenza is for production timeline; delay in monkeypox vaccine is for time to procure supply and to recognize need.

### 2.2. Stage 1

A baseline stage 1 model was constructed for each circulating disease. Target basic reproduction numbers (*r*0) were determined from consulting relevant literature for norovirus and influenza, and a likely maximum number of generations during a monkeypox outbreak (see Table 1). The stage 1 models were designed to achieve the target *r*0 or number of generations. Real life transmission of disease depends on multi-faceted factors: biological, social, structural, and behavioral.^18^ We conceptually break the risk of transmission down into three components: the probability of (1) infectious persons or (2) susceptible persons taking adequate precautions to avoid catching disease, as well as (3) viral shedding (which can be linked to pathogen and illness characteristics, not truly under individual control). The involuntary shedding is separated from behavioral risks for many reasons. It makes sense that a small part of the risk is purely biological not behavioral. Separation lets individual behavior be fully separated from involuntary shedding, while involuntary shedding may be adjusted to vary over the course of illness in future more sophisticated models (the amount of virus shed does varies with the stage of illness in real infections^19^).

In a model run, during each time step and in a random direction, well agents move one step. Well agents move five times further than ill agents (who move 0.2 steps). Each time step, susceptible agents near infectious agents were tested for possible disease transmission. Disease was transmitted if neither side took sufficient precautions and viral shedding was sufficient for transmission. Individuals had baseline take-precautions (TP)% values assigned at the start of model runs. TP is the percentage of the time in which agents took effective precautions against catching disease. TP did not vary in the stage 1 mode, but was important in stage 2 and 3 models when it could change in response to circulating (mis)information. To establish the initial (baseline) stage 1 models, an exercise (described in the next paragraph) was undertaken to estimate the proportion of risk that could be attributed to and reserved for just viral shedding.

TP was assigned to each individual agent, generated stochastically, and assumed to have a normal distribution of values around the population mean, which was preset to 50% for several reasons. Constraining TP to a range only between 0 and 1.0 (but with a fixed central population mean) gave TP the maximum room for change. Also, prespecifying TP allowed us to estimate and specify separately the small proportion of risk of transmission that could be attributed to viral shedding alone (meant to be pathogen-specific and due to the presence of infection, not the risk of transmission due to agents’ good or poor behavior choices). Designating a small separate viral shed risk from behavior seemed desirable because only with fairly extreme precautions (such as wearing personal protection equipment) could the risk of disease transmission be truly reduced to effectively zero. The mechanism of disease transfer was as follows: transmission could happen when susceptible and infectious agents were in close proximity and neither took high enough precautions to avoid transmission. A small proportion of the risk was also linked to viral shedding for each disease. The variations in how disease was transmitted (with and without a separate viral shedding risk) is helpful in showing that our modeling approach can be flexible and adapted for different disease or outbreak conditions.

Table 1 lists other model parameters and assumptions. Between 1% and 2% of agents were infected at simulation start time. Thereafter, agents moved around and tests were made about potentially transmitted disease each time step (1 hour). The model starts at 7am, and agents return “home” to the same location each evening, which means the highest disease transmission risk is with others near their home location. Agent density, grid size, and movement rules were designed such that, in the absence of any disease, the daily contact rate with other agents averaged very close to 11.74 unique others/day (a target drawn from published UK contact rates in non-epidemic situations^20^). Empirically, we found that 1600 agents achieved the target contact rate, on a torus world shape (e.g., going off the bottom meant re-entry at the top), with a visible area measuring 88 × 90 patches that agents could move around on. Incubation periods, assumptions about shedding before or after illness, the duration of active illness and the infectious period, and case fatality rates were drawn from the relevant literature. In Table 1, chances of hospitalization or fatality were plausible, not meant to be definitive. Because health care in the UK is free at the point of use to and urgent care facilities are widely available, we assume that all very ill individuals will seek medical advice. We assume that only very ill individuals will die from disease. Therefore, the models assume that only hospitalized cases ever died and that no transmission occurred after someone was hospitalized due to effective infection control measures. Vaccine efficacy was assumed to be 100%, but vaccine availability varied by disease. Uptake was assumed to be high but not universal (75% of those who could benefit). Hospitalization is the only form of quarantine considered.

### 2.3. Stage 2

The stage 2 model used the same disease and information circulating assumptions as the stage 1 model, but with exacerbation due to spread of misinformation that often reduced taking effective precautions (TP). Recall that the mean TP value was assigned randomly on a normal distribution with a preset mean of 50% at the start. In stage 2, each exposure to misinformation changed individual TP, with limits at 0% and 100%. The possible change in TP (ΔTP) was determined using repeated model runs to achieve a 40% worse (higher) *r*0 for norovirus or influenza, or more generations of transmission (from target 4 to target 7 in 75% of simulations) for monkeypox. The magnitude of change in response to advice was equal whether good or bad advice.^21–23^ΔTP is the key response that individuals have to circulating information (or misinformation). Exposure to “good” advice increases taking precautions; exposure to bad advice decreases taking precautions.

### 2.4. Social contacts and information sharing

Each agent had a list of other agents that they might share information with (their own unique “information bubble”). There were typically 80–230 members of this “bubble” (mean number = 150, to conform with estimates of significant friendship circle size, Dunbar numbers^24,25^). The list of social contacts was created such that (on average) two-thirds of the social contacts had similar propensity (randomly assigned around population mean = 38.9%) to believe misinformation (from experimental and observation data that typically members of the British public believe on an average 38.9% of conspiracy theories that they are exposed to^26^). About 20% of social contacts were located near the agent’s home location; physical proximity made it more likely that disease would be shared with these specific same social contacts with whom (mis)information was shared. In every model run, agents moved around, potentially transmitted disease, and made decisions about whether to share information they had been exposed to. Susceptible agents with lower TP values were at highest risk of acquiring infection.

A total of 138 times per hour a single agent chosen at random was exposed to a piece of information and made a decision (stochastically) whether to share the information onward to a small percentage (2.5%) of their social contacts. The cascades of resulting information (number of times the information passed through unique sharers) were monitored and the model performance in this regard is documented elsewhere.^2^ The frequency of relevant information sharing and the likelihood of sharing advice were both determined empirically so that the resulting information cascades would conform with information sharing patterns reported recently on Twitter for true and false stories.^27^ Distinguishing true from false (good and bad advice in our model) was important, because false stories were observed to be four times more likely to be shared in the Twitter study. In stage 2, the ratio of good:bad advice exposed to agents was 50:50.

### 2.5. Stage 3: intervention strategies

Proposed strategies to fight fake news from the previous literature include the following:

1. provide counter-information that is equally or better evidenced, or more persuasive^28–34^;
2. tax the advertising or tax the profits of products sold via misinformation^35^;
3. drown bad info with good information^35^;
4. regulate information^33^ and possibly impose civil or criminal liabilities,^29^ which could lead to explicit censorship^29,33^;
5. revise financial models available to fake news disseminators (incentives) to stop encouraging the production and sharing of false (or even just very salaciously written) stories over truth and accuracy^27,34,36–39^;
6. labeling (reliability rating or counter-arguments provided) by the news provider ^27,29,33,34^;
7. encourage individuals to actively strive to make their own social filter bubbles more diverse^33^’
8. “immunize” recipients to disregard fake news (education-based strategy).^36,40^

We do not model the effects of intervention strategy 1 because the results are predictable; any changes will be linear responses if good advice increases without a reduction in bad advice or if good and bad advice are equally contagious. Therefore, in stage 3, two strategies for reducing misinformation impacts were tested because their impacts could not be easily foreseen: (1) increasing the proportion of “good advice” that encourages more protective behaviors; and (2) “immunizing” individuals so that they do not respond to or share bad advice. Possible thresholds (to reduce the *r*0 from stage 2 to stage 1 values, or even lower) were explored both for the proportion misinformation adjustment and “immunization” strategies. Stage 3 models were run under stage 2 conditions but with the following modifications and objectives.

- Stage 3.1: reduce bad advice injections from 50% to a value that achieved conditions similar to our stage 1 models (stage 1 target *r*0 or number of generations), as well as the stage 3.2 test of what happened if only 10% of circulating advice is bad advice.
- “Immunize” against bad information (but not immunized against the virus, and still able to react positively to good advice): a percentage of randomly selected agents were selected to be fully resistant (“immunized”) to bad advice. The exact percentage was found empirically, such that (stage 3.3) the stage 1 *r*0 or number of generations was achieved. We also tested (stage 3.4) if “immunizing” 90% of agents could reduce *r*0 below 1.0. “Immunization” also meant no sharing of bad advice. This strategy simulated approaches that were based on education or diversifying social contacts (social filter bubbles).

Statistical differences in the outcomes under each set of modeling assumptions were calculated using Wilcoxon rank sum tests using Stata v. 16.0 software (stage 1 was the reference condition).

## 3. Results

Table 2 shows estimated viral shed risk values found to consistently get the closest to target *r*0s in stage 1 models, when mean TP = 50%, and the change in TP (ΔTP) required upon each information exposure to increase *r*0 by 40% (stage 2 models). Table 3 shows model run results (*r*0, duration of outbreak, final attack rate, and prevalence of disease at peak) for each separate disease. Supplemental files S1–S3 show additional results for alternative model parameters. For all diseases, reducing bad advice from 50% to 40% of all relevant circulating advice changed stage 2 outbreak conditions to stage 1 levels. Similarly, making 20–25% of individuals “immune” to believing or sharing bad advice changed stage 2 outbreak outcomes back to stage 1 levels (with respect to *r*0 or number of generations of transmission, duration of outbreak, peak attack rate, case fatality rate, or final total attack rate).

**Table 2.** Performance metrics and results to generate stage 1–2 models.

	Norovirus	Influenza	Monkeypox
Stage 1. Proportion of transmission risk due to viral shedding (5–95th percentiles for linked *r*0)	8.3% (1.75–2.06)	3.6% (1.27–1.58)	0.8% (0–0.53)
Stage 1. Mean case fatality rate % (CFR, 5–95th percentiles)	n/a	0.70% (0.42–1.03%)	1.04% (0–2.93%)
Stage 2. ΔTP value required to consistently increase *r*0 or gens (5–95th percentiles for linked *r*0)	1.9% (2.44–2.89)	1.05% (1.78–2.32)	1.1% (0–0.89)
Stage 2. Mean case fatality rate % (CFR, 5–95th percentiles)	n/a	0.62% (0.42–1.14%)	1.39% (0–1.68%)

CFR: case fatality rate; gens: generations.

Note: these values most closely enabled meeting the *r*0 or #generations targets (in Table 1). Values in () are range of *r*0 or CFR, 5–95th percentiles, over ≥100 simulations. More detailed results are in the supplemental information.

**Table 3.** Stage 1 (no sharing), stage 2 (outbreak exacerbated by bad advice), and stage 3 (results with intervention strategies). Mean values for given outbreak characteristics, with 5–95th percentiles to indicate range without the most extreme values.

**Stage 1**. No circulating advice					
	*r*0 or #gens	Duration (weeks)	Final attack rate	Peak attack rate	Case fatality rate
Norovirus	1.90 (1.75–2.06)	9.4	78.6%	8.6%	n/a
Influenza	1.46 (1.27–1.58)	13.6	59.2%	14.0%	0.70%
Monkeypox	75th perc #gens = 4	7.3	1.3%	0.98%	1.04%
**Stage 2.** Circulating advice makes outbreak worse, *r*0 increased by 40% or #gens from 4 to 7. Good:bad advice ratio is still 50:50					
	*r*0 or #gens	Duration (weeks)	Final attack rate	Peak attack rate	Case fatality rate
Norovirus	2.66 (2.44–2.89)**	8.7**	91.8%**	10.7%**	n/a
Influenza	2.08 (1.78–2.32) **	14.9**	82.7%**	18.2%**	0.62%**
Monkeypox	75th perc #gens = 7	9.9	2.2%	1.2%	1.39%**
**Stage 3.1.** Strategies to reduce impacts of circulating bad advice in stage 2 conditions: ratio advice needed to revert to stage 1 *r*0 or #gens					
	Good:bad advice ratio	Duration (weeks)	Final attack rate	Peak attack rate	Case fatality rate
Norovirus	59:41*	9.2	79.2%*	8.9%	n/a
Influenza	60:40	14.4	59.0%	13.4%	0.73%
Monkeypox	61:39	7.1	7.3%	1.0%	1.33%
**Stage 3.2.** Stage 2 conditions, but if good:bad advice ratio is 90:10					
	*r*0 or #gens	Duration (weeks)	Final attack rate	Peak attack rate	Case fatality rate
Norovirus	0.99 (0.95–1.03)**	3.9**	21.1%**	3.9%**	n/a
Influenza	0.88 (0.76–0.99) **	5.1**	12.8%**	5.0%**	0.74%**
Monkeypox	75th perc #gens = 3**	5.6**	1.2%*	1.0%	0.96%
**Stage 3.3.** Stage 2 conditions, what % of agents need to be “immunized” against bad advice, needed to revert to stage 1 *r*0 or #gens					
	% “immunized”	Duration (weeks)	Final attack rate	Peak attack rate	Case fatality rate
Norovirus	20%	9.3	78.5%	8.9%	n/a
Influenza	22.5%	15.2**	59.6%	13.1%*	0.42%
Monkeypox	20–40%	6.9–8.4	1.3–1.5%	0.98–1.04%	0.86–1.34%
**Stage 3.4.** Stage 2 conditions, but if 90% of agents are “immunized”					
	*r*0 or #gens	Duration (weeks)	Final attack rate	Peak attack rate	Case fatality rate
Norovirus	1.11 (1.03–1.22)**	5.0**	31.6%**	4.8%**	n/a
Influenza	1.02 (0.88–1.13) **	7.1**	22.4%**	7.5%**	0.15%**
Monkeypox	75th perc #gens = 3*	5.6*	1.2%	1.0%	0.96%

Note: “immunized” means acquired perfect resistance against believing or sharing bad advice, rather than inability to catch norovirus, influenza, or monkeypox. 75th perc #gens is the 75th percentile value (among all eligible simulations) for number of disease transmission generations; 75% of simulations had this number or fewer transmissions. Statistical significance: Wilcoxon rank sum tests with stage 1 outcomes as reference: ** means *p* < 0.01, while * means 0.01 < *p* < 0.05.

We find it interesting to note that even if the strategies are applied relatively drastically and effectively (i.e., quite large reductions in proportion of bad advice circulating or high percentage “immunized” against bad advice), disease spread was not stopped. The results in Table 3 suggest that even if bad advice were only 10% of circulating advice (stage 3.2), *r*0 for norovirus and influenza may reach 1.0, while the number of generations of transmission for monkeypox will be ≥3 in at least 25% of simulations. Similarly, even if 90% of agents are “immune” to bad advice (stage 3.4), outbreak *r*0 (norovirus and influenza) will tend to be ≥1.0 and at least 25% of outbreaks will have ≥3 generations of transmission. The Wilcoxon rank sum tests (Table 3) show that, broadly, outcomes were similar between stage 1 models and stage 3.1 and 3.3 models (which was the objective). Stage 2, 3.2, and 3.4 model outcomes were quite different from stage 1 models (most *p*-values were well below 0.01).

## 4. Discussion

No previous studies have integrated information spread with disease spread to the level of sophistication that we have done. Prior models often considered information spread in disease outbreak development, but information awareness was typically equally available to all agents, and benign at worst. Thus, information spread in the models nearly always led to greater protective measures (such as increasing vaccine uptake or decreasing contact rates^41–50^). Most previous similar disease and awareness spread models had awareness increases that could only happen following physical contact or as a result of global conditions.^42,45,48,50–55^ Our modeling is unusual because information spread was individual and separated from the physical interactions that could transmit disease. Our model is unique and original in attempting to consider the potentially deleterious role of information sharing with stochastic and individually assigned elements. The need for research such as ours has been recognized before.^17,56^

More sophisticated information sharing networks than we tried to create could make these models more credible. There exist more sophisticated models on rumor spread that we could possibly replicate for the information spreading process,^57–59^ and simultaneously merge with existing sophisticated disease spread models. More ambitious models than ours would describe more agents and more complicated movement patterns, such as including flight as a behavior option. Many rumor spreading models have borrowed ideas and methods from epidemiological models,^60,61^ but not many (if any) previous models have integrated both rumor and disease spread as separate but interacting processes into one unified probabilistic model.

This study describes the spread of three viral diseases; misinformation affecting the spread of bacterial diseases could be modeled equally well. The ideas could be applied to non-communicable diseases and health outcomes, but it would be necessary to change the time scale to be much longer to model chronic and lifestyle diseases and how their incidence might change in response to circulating misinformation. A much longer time scale would mean incorporating many other lifestyle factors into the models.

Model construction relied heavily on a small number of existing studies about such factors as number of contact rates, social contacts (i.e.., Dunbar numbers), how much bad or good advice can change behavior, and the propensity to believe in misinformation (the finding that on average, British people believe in 38.9% of conspiracy theories that they are exposed to). More reliably estimating any of these and many of the other factors would also increase the credibility of our results. Our threshold for a “worse” outbreak situation was *r*0 being 40% worse or the number of generations of disease transmission increased from 4 to 7; these thresholds were decided for convenience in this set of demonstration models.

Given our definition of stage 2 as an outbreak “made worse by circulating misinformation,” stage 3.1 modeling concluded for all three diseases that a ratio of about 60:40 good:bad advice circulating would reduce the stage 2 conditions to those of stage 1. The models also suggested that “immunizing” about 20% of the population against misinformation was likely to revert stage 2 to stage 1 conditions (for all diseases, stage 3.3). Since these apparent consistencies could be artefacts of shared model design, tests to explore the true consistency of these findings for multiple diseases would be worthwhile. It is possible that more sophisticated, detailed, or larger models or more flexible modeling software^62^ would facilitate better insights into risk distributions and behavior choices.

There is uncertainty in the reliability of these findings because the models are experimental and have not been tested in real world situations. There is a general lack of reliable quantification for how much misinformation spread impacts real life risk-taking behavior with regard to communicable diseases.

## 5. Conclusions

We applied three stages of modeling (1 = no misinformation spread, 2 = misinformation making outbreaks worse, and 3 = strategies to reduce the influence of misinformation). Our modeling approach and design is adaptable to many different types of diseases. Controlling spread of misinformation or susceptibility to it could reduce communicable disease burdens. Our stage 3.1 modeling found that a ratio of about 60:40 good:bad circulating advice reduced stage 2 conditions to those of stage 1 in three types of disease. “Immunizing” about 20% of the population against misinformation (stage 3.3) was likely to revert stage 2 to stage 1 conditions (for all diseases). The feasibility of implementing these types of strategies (“immunization” or changing the proportions of types of advice in circulation) should be explored. The efficacy of implementing such strategies to fight “fake news” needs to be tested in real world settings, with costs and benefits ideally compared with real world disease reduction.

## Supplemental Material

Simulation_S1 – Supplemental material for Misinformation making a disease outbreak worse: outcomes compared for influenza, monkeypox, and norovirus Click here for additional data file. Supplemental material, Simulation_S1 for Misinformation making a disease outbreak worse: outcomes compared for influenza, monkeypox, and norovirus by Julii Brainard and Paul R Hunter in SIMULATION

Simulation_S2 – Supplemental material for Misinformation making a disease outbreak worse: outcomes compared for influenza, monkeypox, and norovirus Click here for additional data file. Supplemental material, Simulation_S2 for Misinformation making a disease outbreak worse: outcomes compared for influenza, monkeypox, and norovirus by Julii Brainard and Paul R Hunter in SIMULATION

Simulation_S3 – Supplemental material for Misinformation making a disease outbreak worse: outcomes compared for influenza, monkeypox, and norovirus Click here for additional data file. Supplemental material, Simulation_S3 for Misinformation making a disease outbreak worse: outcomes compared for influenza, monkeypox, and norovirus by Julii Brainard and Paul R Hunter in SIMULATION

## References

[1] Brainard J Hunter PR Hall IR . An agent-based model about the effects of fake news on a norovirus outbreak. In: East of England Public Health Conference 2018, Stansted Airport, Stansted, UK, 30 October 2018.

[2] Brainard J Hunter PR Hall I . An agent-based model about the effects of fake news on a norovirus outbreak (under review; also at https://osf.io/cyq6t/?view_only=e422e51b6ab441b0badadd0963821221). 10.1016/j.respe.2019.12.001 32037129

[3] Cava MA Fay KE Beanlands HJ , et al. Risk perception and compliance with quarantine during the SARS outbreak. J Nurs Scholarsh 2005; 37: 343–347. 16396407 10.1111/j.1547-5069.2005.00059.x

[4] Surgeoner BV Chapman BJ Powell DA. University students’ hand hygiene practice during a gastrointestinal outbreak in residence: what they say they do and what they actually do. J Environ Health 2009; 72: 24–29. 19761005

[5] Francis G . Millions of children are sent to school or nursery when ill, poll claims. The Independent, 17 September 2019, https://www.independent.co.uk/news/education/education-news/millions-children-illness-sick-school-nursery-poll-a9108451.html.

[6] Tenkorang EY. Effect of knowledge and perceptions of risks on Ebola-preventive behaviours in Ghana. Int Health 2018; 10: 202–210. 29506203 10.1093/inthealth/ihy009

[7] Vinck P Pham PN Bindu KK , et al. Institutional trust and misinformation in the response to the 2018–19 Ebola outbreak in North Kivu, DR Congo: a population-based survey. Lancet Infect Dis 2019; 19: 529–536. 30928435 10.1016/S1473-3099(19)30063-5

[8] Yamanis T Nolan E Shepler S. Fears and misperceptions of the Ebola response system during the 2014–2015 outbreak in Sierra Leone. PLoS Negl Trop Dis 2016; 10: e0005077. 27755553 10.1371/journal.pntd.0005077 PMC5068712

[9] Blume S. Anti-vaccination movements and their interpretations. Soc Sci Med 2006; 62: 628–642. 16039769 10.1016/j.socscimed.2005.06.020

[10] Hobson-West P. ‘Trusting blindly can be the biggest risk of all’: organised resistance to childhood vaccination in the UK. Sociol Health Illn 2007; 29: 198–215. 17381813 10.1111/j.1467-9566.2007.00544.x

[11] Bass SB Ruzek SB Ward L , et al. If you ask them, will they come? Predictors of quarantine compliance during a hypothetical avian influenza pandemic: results from a statewide survey. Disaster Med Public Health Prep 2010; 4: 135–144. 20526136 10.1001/dmphp.d-09-00052r2

[12] Blendon RJ DesRoches CM Cetron MS , et al. Attitudes toward the use of quarantine in a public health emergency in four countries: the experiences of Hong Kong, Singapore, Taiwan, and the United States are instructive in assessing national responses to disease threats. Health Aff (Millwood) 2006; 25: W15-W25. 10.1377/hlthaff.25.w15 16434437

[13] Wellman MP. Putting the agent in agent-based modeling. Auton Agent Multi Agent Syst 2016; 30: 1175–1189.

[14] Yang C Wilensky U. Netlogo epidem basic model. Evanston, IL: Center for Connected Learning and Computer Based Modeling Northwestern University, 2011.

[15] Willem L Verelst F Bilcke J , et al. Lessons from a decade of individual-based models for infectious disease transmission: a systematic review (2006–2015). BMC Infect Dis 2017; 17: 612. 28893198 10.1186/s12879-017-2699-8 PMC5594572

[16] Federal Select Agent Program. Select agents regulations. Centers for Disease Control and Prevention, Animal and Plant Health Inspection Service, 2017.

[17] Tian D Zheng T. Comparison and analysis of biological agent category lists based on biosafety and biodefense. PLoS One 2014; 9: e101163. 24979754 10.1371/journal.pone.0101163 PMC4076228

[18] Swift L Hunter PR Lees AC , et al. Wildlife trade and the emergence of infectious diseases. EcoHealth 2007; 4: 25.

[19] Lau LL Cowling BJ Fang VJ , et al. Viral shedding and clinical illness in naturally acquired influenza virus infections. J Infect Dis 2010; 201: 1509–1516. 20377412 10.1086/652241 PMC3060408

[20] Mossong J Hens N Jit M , et al. Social contacts and mixing patterns relevant to the spread of infectious diseases. PLoS Med 2008; 5: e74. 18366252 10.1371/journal.pmed.0050074 PMC2270306

[21] Gallagher KM Updegraff JA. Health message framing effects on attitudes, intentions, and behavior: a meta-analytic review. Ann Behav Med 2011; 43: 101–116. 10.1007/s12160-011-9308-7 21993844

[22] Lithopoulos A Bassett-Gunter RL Martin Ginis KA , et al. The effects of gain-versus loss-framed messages following health risk information on physical activity in individuals with multiple sclerosis. J Health Commun 2017; 22: 523–531. 28481157 10.1080/10810730.2017.1318983

[23] O’Keefe DJ Jensen JD. The advantages of compliance or the disadvantages of noncompliance? A meta-analytic review of the relative persuasive effectiveness of gain-framed and loss-framed messages. Ann Int Commun Assoc 2006; 30: 1–43.

[24] Dunbar RI Arnaboldi V Conti M , et al. The structure of online social networks mirrors those in the offline world. Soc Network 2015; 43: 39–47.

[25] Mac Carron P Kaski K Dunbar R . Calling Dunbar’s numbers. Soc Network 2016; 47: 151–155.

[26] Brotherton R French CC Pickering AD. Measuring belief in conspiracy theories: the generic conspiracist beliefs scale. Front Psychol 2013; 4: 279. 23734136 10.3389/fpsyg.2013.00279 PMC3659314

[27] Vosoughi S Roy D Aral S. The spread of true and false news online. Science 2018; 359: 1146–1151. 29590045 10.1126/science.aap9559

[28] Jervelund SS. How social media is transforming the spreading of knowledge: Implications for our perceptions concerning vaccinations and migrant health. Scand J Public Health 2018; 46: 167–169. 29569533 10.1177/1403494818760139

[29] Lazer DM Baum MA Benkler Y , et al. The science of fake news. Science 2018; 359: 1094–1096. 29590025 10.1126/science.aao2998

[30] Oyeyemi SO Gabarron E Wynn R. Ebola, Twitter, and misinformation: a dangerous combination? Br Med J 2014; 349: g6178. 25315514 10.1136/bmj.g6178

[31] Haer T Botzen WW Aerts JC. The effectiveness of flood risk communication strategies and the influence of social networks—insights from an agent-based model. Environ Sci Policy 2016; 60: 44–52.

[32] Berger J Milkman KL. What makes online content viral? J Marketing Res 2012; 49: 192–205.

[33] Garrett RK. The “echo chamber” distraction: disinformation campaigns are the problem, not audience fragmentation. J Appl Res Memory Cognit 2017; 6: 370–376.

[34] Lyons T. Replacing disputed flags with related articles, https://newsroom.fb.com/news/2017/12/news-feed-fyi-updates-in-our-fight-against-misinformation/ (2017, accessed 24 July 2018).

[35] Glaeser EL Ujhelyi G. Regulating misinformation. J Public Econ 2010; 94: 247–257.

[36] MSNBC. “Finding Foothold in the Age of Trump” Television broadcast on 10 April 2018.

[37] Allcott H Gentzkow M. Social media and fake news in the 2016 election. J Econ Perspect 2017; 31: 211–236.

[38] Ball J . Post-truth: how bullshit conquered the world. Biteback Publishing: London, UK, 2017.

[39] Shane S. From headline to photograph, a fake news masterpiece. The New York Times, 18 January 2017, https://www.nytimes.com/2017/01/18/us/fake-news-hillary-clinton-cameron-harris.html.

[40] Rochlin N. Fake news: belief in post-truth. Library Hi Tech 2017; 35: 386–392.

[41] Andrews MA Bauch CT. Disease interventions can interfere with one another through disease-behaviour interactions. PLoS Comput Biol 2015; 11: e1004291. 26047028 10.1371/journal.pcbi.1004291 PMC4457811

[42] Bisset KR Feng X Marathe M , et al. Modeling interaction between individuals, social networks and public policy to support public health epidemiology. In: Rossetti MD Hill RR Johansson B , et al. (eds) Winter Simulation Conference, Institute of Electrical and Electronics Engineers, Austin, TX, USA, 13–16 December 2009, pp.2020–2031.

[43] Chen F Jiang M Rabidoux S , et al. Public avoidance and epidemics: insights from an economic model. J Theor Biol 2011; 278: 107–119. 21419135 10.1016/j.jtbi.2011.03.007

[44] d’Onofrio A Manfredi P. Information-related changes in contact patterns may trigger oscillations in the endemic prevalence of infectious diseases. J Theor Biol 2009; 256: 473–478. 18992258 10.1016/j.jtbi.2008.10.005

[45] Durham DP Casman EA. Incorporating individual health-protective decisions into disease transmission models: a mathematical framework. J Royal Soc Interf 2011; 9: rsif20110325. 10.1098/rsif.2011.0325 PMC3262418 21775324

[46] Guo D Li KC Peters TR , et al. Multi-scale modeling for the transmission of influenza and the evaluation of interventions toward it. Sci Rep 2015; 5: 8980. 25757402 10.1038/srep08980 PMC4355742

[47] Hatzopoulos V Taylor M Simon PL , et al. Multiple sources and routes of information transmission: implications for epidemic dynamics. Math Biosci 2011; 231: 197–209. 21397611 10.1016/j.mbs.2011.03.006

[48] Mao L. Predicting self-initiated preventive behavior against epidemics with an agent-based relative agreement model. J Artif Soc Simulat 2015; 18: 6.

[49] Smith MC Broniatowski DA. Modeling influenza by modulating flu awareness. Cham: Springer International Publishing, 2016, pp.262–271.

[50] Zhang H-F Xie J-R Chen H-S , et al. Impact of asymptomatic infection on coupled disease-behavior dynamics in complex networks. EPL (Europhys Lett) 2016; 114: 38004.

[51] Epstein JM Parker J Cummings D , et al. Coupled contagion dynamics of fear and disease: mathematical and computational explorations. PLoS One 2008; 3: e3955. 19079607 10.1371/journal.pone.0003955 PMC2596968

[52] Fenichel EP Castillo-Chavez C Ceddia MG , et al. Adaptive human behavior in epidemiological models. Proc Natl Acad Sci 2011; 108: 6306–6311. 21444809 10.1073/pnas.1011250108 PMC3076845

[53] Fu F Rosenbloom DI Wang L , et al. Imitation dynamics of vaccination behaviour on social networks. Proc R Soc Lon B Biol Sci 2011; 278: 42–49. 10.1098/rspb.2010.1107 PMC2992723 20667876

[54] Nunez F Ravello C Urbina H , et al. A rule-based model of a hypothetical zombie outbreak: Insights on the role of emotional factors during behavioral adaptation of an artificial population. arXiv preprint arXiv:12104469 2012.

[55] Tully S Cojocaru M Bauch CT. Coevolution of risk perception, sexual behaviour, and HIV transmission in an agent-based model. J Theor Biol 2013; 337: 125–132. 23988796 10.1016/j.jtbi.2013.08.014

[56] Fajebe A. Computational modeling of spontaneous behavior changes and infectious disease spread. PhD Thesis, Georgia Institute of Technology, 2016.

[57] Lukasik M Cohn T Bontcheva K . Point process modelling of rumour dynamics in social media. In: Zong C Strube M (eds) Proceedings of the 53rd annual meeting of the Association for Computational Linguistics and the 7th International Joint Conference on Natural Language Processing (Volume 2: short papers), Beijing, China, 26–31 July 2015, pp.518–523. Red Hook, New York: Association for Computational Linguistics.

[58] Wang A Wu W Chen J. A novel rumour propagation model on social networks. Int J Sensor Network 2017; 25: 126–133.

[59] Nekovee M Moreno Y Bianconi G , et al. Theory of rumour spreading in complex social networks. Physica A Stat Mech Appl 2007; 374: 457–470.

[60] Pearce CE. The exact solution of the general stochastic rumour. Math Comput Model 2000; 31: 289–298.

[61] Huo L Huang P Guo C-x. Analyzing the dynamics of a rumor transmission model with incubation. Discrete Dynam Nat Soc 2012; 2012, 10.1155/2012/328151.

[62] Jenness SM Goodreau SM Morris M. EpiModel: an R package for mathematical modeling of infectious disease over networks. J Stat Software 2018; 84, doi:10.18637/jss.v084.i08. PMC5931789 29731699

[63] Gaythorpe K Trotter CL Lopman B , et al. Norovirus transmission dynamics: a modelling review. Epidemiol Infect 2018; 146: 147–158. 29268812 10.1017/S0950268817002692 PMC5851036

[64] Biggerstaff M Cauchemez S Reed C , et al. Estimates of the reproduction number for seasonal, pandemic, and zoonotic influenza: a systematic review of the literature. BMC Infect Dis 2014; 14: 480. 25186370 10.1186/1471-2334-14-480 PMC4169819

[65] Blumberg S Lloyd-Smith JO. Inference of R0 and transmission heterogeneity from the size distribution of stuttering chains. PLoS Comput Biol 2013; 9: e1002993. 23658504 10.1371/journal.pcbi.1002993 PMC3642075

[66] Sklenovská N Van Ranst M. Emergence of monkeypox as the most important orthopoxvirus Infection in humans. Front Publ Health 2018; 6: Article 241. 10.3389/fpubh.2018.00241 PMC6131633 30234087

[67] Centers for Disease Control and Prevention. Norovirus in healthcare facilities fact sheet, https://www.cdc.gov/hai/pdfs/norovirus/229110-ANoroCaseFactSheet508.pdf (2011, accessed 24 October 2018).

[68] Lee RM Lessler J Lee RA , et al. Incubation periods of viral gastroenteritis: a systematic review. BMC Infect Dis 2013; 13: 446. 24066865 10.1186/1471-2334-13-446 PMC3849296

[69] Centers for Disease Control and Prevention. Clinical signs and symptoms of influenza, https://www.cdc.gov/flu/professionals/acip/clinical.htm (2008, accessed 24 October 2018).

[70] McCollum AM Damon IK. Human monkeypox. Clin Infect Dis 2013; 58: 260–267. 24158414 10.1093/cid/cit703 PMC5895105

[71] Devasia T Lopman B Leon J , et al. Association of host, agent and environment characteristics and the duration of incubation and symptomatic periods of norovirus gastroenteritis. Epidemiol Infect 2015; 143: 2308–2314. 25483148 10.1017/S0950268814003288 PMC8845055

[72] Mesquita J Nascimento MS. A foodborne outbreak of norovirus gastroenteritis associated with a Christmas dinner in Porto, Portugal, December 2008. Eurosurveillance 2009; 14: 19355. 19883537

[73] Centers for Disease Control and Prevention. The symptoms of norovirus, https://www.cdc.gov/norovirus/about/symptoms.html (2015, accessed 24 October 2018).

[74] Hadler JL Konty K McVeigh KH , et al. Case fatality rates based on population estimates of influenza-like illness due to novel H1N1 influenza: New York City, May–June 2009. PLoS One 2010; 5: e11677. 20657738 10.1371/journal.pone.0011677 PMC2908148

[75] World Health Organization. Monkeypox, http://www.who.int/news-room/fact-sheets/detail/monkeypox (2018, accessed 24 October 2018).

[76] Gerdil C. The annual production cycle for influenza vaccine. Vaccine 2003; 21: 1776–1779. 12686093 10.1016/s0264-410x(03)00071-9

[77] Centers for Disease Control and Prevention. Smallpox vaccine guidance, https://www.cdc.gov/poxvirus/monkeypox/clinicians/smallpox-vaccine.html (2015, accessed 24 October 2018).

[78] Centers for Disease Control and Prevention. Key facts about seasonal flu vaccine, https://www.cdc.gov/flu/protect/keyfacts.htm (2018, accessed 24 October 2018).

