# Supplementary material for: Misinformation making a disease outbreak worse: outcomes compared for influenza, monkeypox, and norovirus
Source: Simulation. 2020 Apr;96(4):365–74. doi: 10.1177/0037549719885021 (PMC8282656; doi:10.1177/0037549719885021)
Supplement: Simulation_S1 – Supplemental material for Misinformation making a disease outbreak worse: outcomes compared for influenza, monkeypox, and norovirus [file Simulation_S1.pdf]

Misinformation making a disease outbreak worse: Outcomes compared for influenza, monkeypox and norovirus, S1: Additional results for Influenza

ADDITIONAL RESULTS FOR INFLUENZA

Results of exercise to find proportion of risk attributable to viral shedding.

viralshedrisk	Mean TP	Mean r0	duration (wks)	AR	CFR	maxill	# of iterations	r0min	r0max
2.90%	50%	1.23695	13.8	42.1%	0.639%	8.6%	10	1.05	1.36
3.00%	50%	1.26338	15.9	45.2%	0.581%	8.9%	10	1.17	1.40
3.10%	50%	1.327	14.5	49.4%	0.684%	10.6%	10	1.11	1.50
3.20%	50%	1.31262	14.7	48.9%	0.766%	9.4%	10	1.16	1.43
3.30%	50%	1.38818	14.6	53.9%	0.669%	12.2%	30	1.17	1.61
3.40%	50%	1.42864	14.4	57.5%	0.634%	12.2%	30	1.32	1.53
3.50%	50%	1.45276	15.2	58.6%	0.696%	12.6%	100	1.24	1.61
3.60%	50%	1.45731	13.6	59.2%	0.697%	14.0%	100	1.27	1.58
3.70%	50%	1.5013	14.0	61.7%	0.730%	14.4%	100	1.30	1.64
3.80%	50%	1.53457	13.8	63.4%	0.753%	15.2%	100	1.34	1.70
3.90%	50%	1.5624	14.2	64.9%	0.685%	15.3%	100	1.35	1.72
4.00%	50%	1.6427	13.4	69.0%	0.736%	16.5%	30	1.48	1.77
4.10%	50%	1.6316	13.7	68.5%	0.681%	17.3%	30	1.43	1.78
4.20%	50%	1.64493	14.9	68.9%	0.651%	16.9%	30	1.48	1.79
4.30%	50%	1.66889	14.4	69.6%	0.673%	16.8%	10	1.41	1.86

Pink and Purple fill: Parameters that best met target objectives
TP: presumed mean % of time that exposed individuals take effective precautions to avoid disease.
r0: basic reproduction number
AR: Attack rate
CFR: Case fatality rate
maxIll: Maximum number of persons ill at any one time

Other model assumptions as described in main text.

Misinformation making a disease outbreak worse: Outcomes compared for influenza, monkeypox and norovirus, S1: Additional results for Influenza

Results of exercise to find change in taking precautions (ΔTP) that exacerbated outbreak using pre-specified indicators

deltaTP	Mean r0	medianr0	r0 5%tile	r0 95%tile	duration (wks)	AR	CFR	Peak AR	# iterations
0.6%	1.8413	1.820487	1.61012	2.036392	15.4	76.4%	0.49%	15.5%	30
0.65%	1.847073	1.872104	1.628347	2.036083	16.2	76.3%	0.53%	14.5%	30
0.70%	1.897948	1.903299	1.75542	2.017648	15.1	78.3%	0.55%	17.5%	30
0.75%	1.940064	1.924411	1.705578	2.222077	14.9	79.2%	0.57%	17.4%	60
0.80%	1.9194	1.940195	1.633368	2.104002	14.7	78.5%	0.58%	17.4%	80
0.85%	1.986552	1.980394	1.703666	2.249744	14.7	80.3%	0.57%	17.9%	100
0.90%	1.99107	1.995705	1.738602	2.208379	14.6	80.6%	0.60%	17.2%	100
0.95%	2.025176	2.029149	1.724042	2.277316	15.0	81.5%	0.57%	18.2%	100
1.00%	2.033176	2.024844	1.858305	2.248283	14.2	81.8%	0.56%	18.4%	100
1.05%	2.078659	2.095103	1.781198	2.31627	14.9	82.7%	0.62%	18.2%	100
1.10%	2.080042	2.090032	1.7777	2.370871	14.0	82.8%	0.54%	18.4%	100
1.15%	2.102982	2.125724	1.860752	2.310989	13.9	83.3%	0.57%	18.9%	100
1.20%	2.127492	2.136538	1.850106	2.358577	13.2	83.9%	0.60%	19.7%	100
1.25%	2.159474	2.17899	1.87669	2.393012	13.6	84.4%	0.58%	19.3%	100
1.30%	2.187367	2.172252	1.958251	2.452679	13.4	85.0%	0.48%	20.1%	20
1.35%	2.218797	2.225172	2.018652	2.478924	13.4	85.6%	0.62%	19.5%	10
1.40%	2.220509	2.213158	2.047423	2.41053	13.4	85.8%	0.58%	19.7%	10
1.45%	2.271251	2.235715	2.105149	2.493754	13.6	86.7%	0.61%	19.8%	10

Pink or Purple fill: Parameters that best met target objectives

TP: presumed mean % of time that exposed individuals take effective precautions to avoid disease.
r0: basic reproduction number
AR: Attack rate
CFR: Case fatality rate
maxIll: Maximum number of persons ill at any one time

Other model assumptions as described in main text.

Misinformation making a disease outbreak worse: Outcomes compared for influenza, monkeypox and norovirus, S1: Additional results for Influenza

Impacts on outbreak parameters from varying % of circulating advice that is bad advice.

%BA	Mean r0	r0 5%tile	r0 95%tile	Duration (wks)	Final AR	CFR	Peak AR	# of iterations
32.0%	1.198539	1.079773	1.31704	9.9	39.6%	0.44%	11.0%	10
33.00%	1.16735	1.085505	1.223602	10.2	36.8%	0.65%	10.4%	10
34.00%	1.204356	1.009126	1.377533	9.9	39.7%	0.91%	12.0%	10
35.00%	1.295781	1.19286	1.407595	10.9	47.8%	0.61%	12.2%	10
36.00%	1.293564	1.161435	1.448734	11.6	47.4%	0.69%	12.4%	100
37.00%	1.330352	1.186886	1.484488	11.8	52.5%	0.67%	12.9%	100
38.00%	1.356305	1.207999	1.498873	12.3	52.5%	0.67%	12.9%	100
39.00%	1.393145	1.229517	1.54894	12.7	54.9%	0.70%	13.5%	100
40.00%	1.459654	1.265615	1.613018	14.4	59.0%	0.73%	13.4%	100
41.00%	1.514867	1.352087	1.685933	14.5	62.4%	0.69%	13.8%	100
42.00%	1.541547	1.390222	1.709729	14.2	63.9%	0.69%	14.5%	100
43.00%	1.728418	1.414188	1.91006	14.8	68.2%	0.70%	15.1%	100
44.00%	1.728418	1.414188	1.91006	12.3	71.9%	0.72%	17.1%	10
45.00%	1.746742	1.655819	1.873223	14.7	73.2%	0.60%	15.6%	10
46.00%	1.718664	1.586687	1.818247	14.9	72.5%	0.69%	15.4%	10
47.00%	1.958176	1.804724	2.16928	15.7	80.0%	0.69%	17.2%	10
48.00%	1.91463	1.767755	2.132593	13.6	78.6%	0.80%	16.0%	10
49.00%	2.054574	1.746902	2.227619	13.6	82.1%	0.74%	18.3%	10
10.0%	0.877875	0.755375	0.993021	5.1	12.8%	0.74%	5.0%	100

Pink or Purple fill: Parameters that best met target objectives

%BA: tested %s of total circulating advice that is bad advice
r0: basic reproduction number
AR: Attack rate
CFR: Case fatality rate

Other model assumptions as described in main text.

Misinformation making a disease outbreak worse: Outcomes compared for influenza, monkeypox and norovirus, S1: Additional results for Influenza

Impacts on outbreak parameters from immunising specified %s of population against believing or circulating bad advice

%imm	Mean r0	r0 5%tile	r0 95%tile	Duration (wks)	Final AR	CFR	Peak AR	# of iterations
5.0%	1.912	1.675	2.091	14.2	78.5%	0.53%	17.9%	10
7.5%	1.785	1.565	2.008	14.2	74.3%	0.47%	17.3%	30
10.0%	1.775	1.623	1.968	15.3	74.2%	0.48%	16.3%	30
12.5%	1.687	1.506	1.821	14.2	70.9%	0.49%	15.8%	30
15.0%	1.653	1.478	1.787	15.2	69.2%	0.44%	14.8%	30
17.5%	1.571	1.419	1.731	14.8	65.4%	0.42%	14.4%	30
20.0%	1.543	1.425	1.681	14.5	64.1%	0.46%	14.2%	30
22.5%	1.469	1.310	1.628	15.2	59.6%	0.42%	13.1%	100
25.0%	1.447	1.271	1.596	13.3	58.6%	0.39%	14.5%	100
30.0%	1.372	1.223	1.496	13.1	53.1%	0.43%	13.1%	30
35.0%	1.279	1.150	1.395	13.9	46.4%	0.29%	10.8%	30
40.0%	1.233	1.124	1.364	11.0	42.6%	0.29%	11.4%	30
45.0%	1.207	1.053	1.334	10.8	40.2%	0.27%	11.0%	30
50.0%	1.151	1.063	1.254	10.5	35.3%	0.21%	10.1%	30
60.0%	1.122	0.988	1.226	9.4	32.3%	0.24%	9.3%	30
70.0%	1.059	0.950	1.149	8.3	26.3%	0.15%	8.2%	30
80.0%	1.017	0.872	1.137	7.4	23.1%	0.18%	7.6%	30
90.0%	1.013	0.881	1.126	7.0	22.4%	0.15%	7.5%	100

Pink or Purple fill: Parameters that best met target objectives

%imm: %s of population ‘immunised’ so that they neither believe nor share bad advice
r0: basic reproduction number
AR: Attack rate
CFR: Case fatality rate

Other model assumptions as described in main text.
