# Supplementary material for: Misinformation making a disease outbreak worse: outcomes compared for influenza, monkeypox, and norovirus
Source: Simulation. 2020 Apr;96(4):365–74. doi: 10.1177/0037549719885021 (PMC8282656; doi:10.1177/0037549719885021)
Supplement: Simulation_S2 – Supplemental material for Misinformation making a disease outbreak worse: outcomes compared for influenza, monkeypox, and norovirus [file Simulation_S2.pdf]

Misinformation making a disease outbreak worse: Outcomes compared for influenza, monkeypox and norovirus, S2: Additional results for Monkeypox

ADDITIONAL RESULTS FOR MONKEYPOX

Results of exercise to find proportion of risk attributable to viral shedding.

Viral shed risk	maxGens (75th centile)	r0	Duration (wks)	AR	CFR	maxill	# of iterations	gens.5th	gens.95th
0.10%	1.00	0.01	3.1	1.0%	1.274%	0.97%	10	1.0	2.0
0.20%	1.75	0.03	3.8	1.2%	0.541%	1.11%	10	1.0	2.6
0.30%	1.75	0.02	3.8	1.1%	0.565%	1.08%	10	1.0	2.6
0.40%	2.00	0.06	4.8	1.2%	1.604%	1.08%	10	1.0	3.0
0.50%	3.00	0.10	5.4	1.1%	0.749%	0.99%	112	1.0	5.0
0.60%	3.00	0.12	5.8	1.2%	1.117%	0.98%	150	1.0	5.0
0.70%	3.00	0.13	6.4	1.2%	0.779%	0.99%	150	1.0	5.0
0.80%	4.00	0.17	7.3	1.3%	1.040%	0.98%	160	1.0	8.0
0.90%	4.00	0.22	8.1	1.4%	1.122%	1.01%	160	1.0	8.0
1.00%	6.00	0.26	9.5	1.6%	1.456%	1.01%	160	1.0	10.0
1.10%	6.00	0.27	9.9	1.7%	1.375%	1.02%	60	1.0	9.1
1.20%	7.00	0.37	12.0	2.1%	1.412%	1.07%	60	1.0	11.1
1.30%	10.00	0.39	13.3	2.3%	1.674%	1.06%	60	2.0	12.0
1.40%	8.00	0.46	14.1	2.1%	1.484%	0.95%	10	1.9	10.6
1.50%	11.00	0.46	15.1	3.4%	1.487%	1.25%	10	1.5	13.1
1.60%	7.50	0.42	13.6	2.3%	1.340%	1.09%	10	2.5	11.6
1.70%	13.75	0.70	22.2	5.7%	2.093%	1.56%	10	4.4	14.0

Pink and Purple fill: Parameters that best met target objectives

Viral shed risk: test % of total risk attributable to shedding virus (exclusive of other risk factors)

maxGens (75th centile): maximum number of generations, 75th percentile of all observed model runs
Purpose of this indicator is to find likely parameters when most observed outbreaks will have this maximum number of generations of transmission

TP (mean % of time that exposed individuals take effective precautions to avoid disease): 50% in all Stage 1 runs
r0: basic reproduction number
AR: Attack rate
CFR: Case fatality rate
maxIll: Maximum number of persons ill at any one time
gens.5th: 5th percentile, number of generations of transmission for all relevant iterations
gens 95th: 95th percentile of number of generations of transmission for all relevant iterations

Other model assumptions as described in main text.

Misinformation making a disease outbreak worse: Outcomes compared for influenza, monkeypox and norovirus, S2: Additional results for Monkeypox

Results of exercise to find change in taking precautions (ΔTP) that exacerbated outbreak using pre-specified indicators

Delta TP	maxGens (75th centile)	Median # of Gens	r0	Duration (wks)	AR	CFR	Peak AR	# of iterations
0.1%	3.0	2	0.15	6.7	1.2%	1.21%	1.0%	50
0.2%	4.0	3	0.22	8.5	1.4%	1.26%	0.0%	90
0.3%	4.0	3	0.19	8.3	1.4%	1.40%	1.0%	90
0.4%	4.0	2	0.20	8.5	1.5%	1.39%	1.0%	200
0.5%	5.0	3	0.23	8.8	1.5%	1.58%	1.0%	201
0.6%	5.0	3	0.23	9.8	1.8%	1.42%	1.1%	211
0.7%	5.0	3	0.23	8.9	1.7%	1.48%	1.1%	211
0.8%	5.0	3	0.26	9.5	1.8%	1.35%	1.1%	361
0.9%	5.0	3	0.26	9.5	2.0%	1.61%	1.2%	470
1.0%	6.0	3	0.28	9.8	2.1%	1.37%	1.1%	471
1.1%	7.0	3	0.28	9.9	2.2%	1.36%	1.2%	680
1.2%	9.3	3	0.32	10.5	2.5%	1.55%	1.3%	420
1.3%	10.0	3	0.32	10.6	2.8%	1.51%	1.4%	241
1.4%	10.0	3	0.32	10.5	2.8%	1.42%	1.4%	201
1.5%	11.0	4	0.43	12.8	3.9%	1.60%	1.6%	50
1.6%	11.0	2.5	0.38	11.8	3.3%	1.70%	1.5%	50
1.7%	11.0	4	0.37	12.4	3.0%	1.51%	1.4%	50
1.8%	11.0	5	0.46	14.1	3.6%	1.20%	1.5%	20

Pink or Purple fill: Parameters that best met target objectives

maxGens (75th centile): maximum number of generations, 75th percentile of all observed model runs
Purpose of this indicator is to find likely parameters when most observed outbreaks will have this maximum number of generations of transmission

TP: presumed mean % of time that exposed individuals take effective precautions to avoid disease.
Delta TP: candidate changes in TP with each exposure to good or bad advice
r0: basic reproduction number
AR: Attack rate
CFR: Case fatality rate
maxIll: Maximum number of persons ill at any one time

Other model assumptions as described in main text.

Misinformation making a disease outbreak worse: Outcomes compared for influenza, monkeypox and norovirus, S2: Additional results for Monkeypox

Impacts on outbreak parameters from varying % of circulating advice that is bad advice.

%BA	maxGens (75th centile)	Median Gens	r0	Duration (wks)	AR	CFR	Peak AR	# of iterations
10.0%	3.00	2	0.11	5.2	1.11%	0.96%	0.98%	100
20.0%	3.00	2	0.12	5.6	1.15%	0.95%	0.98%	40
30.0%	3.00	2	0.13	5.9	1.15%	0.95%	0.98%	40
31.0%	3.00	2	0.13	6.0	1.23%	0.80%	1.04%	70
32.0%	3.00	2	0.14	6.0	1.22%	1.17%	1.03%	70
33.0%	3.00	2.5	0.11	6.6	1.18%	1.75%	0.95%	70
34.0%	3.00	2	0.11	5.7	1.13%	1.51%	0.97%	70
35.0%	3.25	2	0.16	6.6	1.19%	1.74%	0.95%	100
36.0%	4.00	2	0.17	1.8	1.25%	1.80%	0.99%	225
37.0%	4.00	2	0.16	6.9	1.26%	1.17%	0.99%	225
38.0%	4.00	2	0.17	7.1	1.35%	1.10%	1.04%	195
39.0%	4.00	2	0.17	7.3	1.32%	1.33%	0.99%	328
40.0%	4.00	3	0.19	7.5	1.35%	1.21%	1.01%	203
41.0%	4.00	2	0.18	7.3	1.29%	1.43%	0.99%	203
42.0%	4.00	3	0.19	8.1	1.42%	1.43%	1.02%	203
43.0%	5.00	3	0.23	9.2	1.63%	1.53%	1.04%	203
44.0%	5.00	3	0.25	9.7	1.70%	1.50%	1.05%	201
45.0%	5.00	3	0.24	9.1	1.72%	1.47%	1.06%	203
46.0%	5.00	3	0.24	9.3	1.76%	1.15%	1.07%	300
47.0%	6.00	3	0.27	9.8	2.03%	1.43%	1.15%	401
48.0%	9.00	3	0.30	10.6	2.22%	1.35%	1.17%	500
49.0%	9.00	3	0.30	10.5	2.38%	1.38%	1.23%	501

Pink or Purple fill: Parameters that best met target objectives

%BA: tested %s of total circulating advice that is bad advice
r0: basic reproduction number
AR: Attack rate
CFR: Case fatality rate

Other model assumptions as described in main text.

Misinformation making a disease outbreak worse: Outcomes compared for influenza, monkeypox and norovirus, S2: Additional results for Monkeypox

Impacts on outbreak parameters from immunising specified %s of population against believing or circulating bad advice

imm%	maxGens (75th centile)	Median # of Gens	r0	wks	AR	CFR	Peak AR	# of iterations
5.0%	7.0	3	0.29	10.1	2.2%	1.56%	1.17%	200
10.0%	6.0	3	0.28	10.0	1.9%	1.35%	1.09%	300
15.0%	5.0	3	0.24	9.2	1.7%	0.00%	1.06%	300
20.0%	4.0	3	0.21	8.4	1.5%	1.17%	1.04%	300
25.0%	4.0	2	0.18	7.6	1.3%	1.12%	0.98%	200
30.0%	4.0	2	0.16	7.0	1.3%	1.34%	1.01%	200
35.0%	4.0	3	0.16	6.9	1.3%	0.86%	1.02%	200
40.0%	4.0	2	0.17	7.1	1.3%	1.25%	1.00%	200
45.0%	3.0	2	0.13	5.9	1.2%	1.13%	0.98%	200
50.0%	3.0	2	0.14	6.2	1.2%	1.24%	1.03%	70
55.0%	3.0	2	0.16	6.1	1.2%	1.59%	0.98%	70
60.0%	3.0	2	0.15	6.4	1.2%	1.77%	0.99%	70
65.0%	3.0	2	0.14	6.2	1.2%	1.46%	1.02%	70
70.0%	3.0	2	0.14	5.9	1.2%	1.37%	1.05%	70
75.0%	3.0	2	0.11	5.4	1.2%	1.27%	1.04%	70
80.0%	3.0	2	0.13	5.9	1.2%	0.98%	1.02%	70
85.0%	3.0	2	0.13	5.6	1.1%	0.86%	0.97%	70
90.0%	3.0	2	0.12	5.6	1.2%	0.96%	1.02%	200

Pink or Purple fill: Parameters that best met target objectives

%imm: %s of population ‘immunised’ so that they neither believe nor share bad advice
r0: basic reproduction number
AR: Attack rate
CFR: Case fatality rate

Other model assumptions as described in main text.
