# Supplementary material for: Misinformation making a disease outbreak worse: outcomes compared for influenza, monkeypox, and norovirus
Source: Simulation. 2020 Apr;96(4):365–74. doi: 10.1177/0037549719885021 (PMC8282656; doi:10.1177/0037549719885021)
Supplement: Simulation_S3 – Supplemental material for Misinformation making a disease outbreak worse: outcomes compared for influenza, monkeypox, and norovirus [file Simulation_S3.pdf]

Misinformation making a disease outbreak worse: Outcomes compared for influenza, monkeypox and norovirus, S3: Additional results for Norovirus

ADDITIONAL RESULTS FOR NOROVIRUS

Results of exercise to find proportion of risk attributable to viral shedding.

Viral shed risk	r0	Duration (wks)	AR	maxill	# of iterations	r0.5%	r0.95%
7.6%	1.80187	9.9	75.5%	8.2%	40	1.67	1.91
7.7%	1.83601	10.0	76.5%	8.2%	40	1.66	1.98
7.8%	1.83538	9.9	76.5%	8.2%	40	1.70	1.97
7.9%	1.84607	9.9	76.9%	8.2%	100	1.72	2.02
8.0%	1.84753	10.1	77.0%	8.3%	100	1.70	1.99
8.1%	1.86388	10.2	77.4%	8.5%	100	1.66	2.02
8.2%	1.87757	9.6	77.8%	8.5%	100	1.71	2.05
8.3%	1.9025	9.4	78.6%	8.6%	100	1.75	2.06
8.4%	1.93665	10.0	79.6%	9.0%	100	1.78	2.08
8.5%	1.95214	9.4	80.1%	9.1%	100	1.80	2.13
8.6%	1.95134	9.5	80.0%	9.1%	100	1.76	2.09
8.7%	1.97349	9.4	80.6%	9.2%	100	1.81	2.14
8.8%	1.979	9.1	80.7%	9.3%	100	1.81	2.15
8.9%	2.00695	9.6	81.4%	9.5%	100	1.83	2.18
9.0%	2.02719	9.2	81.9%	9.3%	100	1.91	2.15
9.1%	2.03017	9.2	82.0%	9.6%	100	1.88	2.18
9.2%	2.04248	9.0	82.3%	9.3%	100	1.86	2.22
9.3%	2.06827	9.7	82.9%	10.0%	40	1.90	2.21
9.4%	2.0771	9.0	83.2%	10.0%	40	1.96	2.23

Pink and Purple fill: Parameters that best met target objectives

TP: preset to 50% at model start, presumed mean % of time that exposed individuals take effective precautions to avoid disease.
Viral shed risk: % of risk of transmission that is attributable to viral shedding alone
r0: basic reproduction number
AR: Attack rate
CFR: Case fatality rate
maxIll: Maximum number of persons ill at any one time
r0.5% & r0.95%: 5%tile & 95%tiles, r0 values across all iterations

Other model assumptions as described in main text.

Misinformation making a disease outbreak worse: Outcomes compared for influenza, monkeypox and norovirus, S3: Additional results for Norovirus

Results of exercise to find change in taking precautions (ΔTP) that exacerbated outbreak using pre-specified indicators

Delta TP	Mean r0	Median r0	Duration (wks)	AR	Peak AR	# of iterations
1.0%	2.278413	2.275761	9.1	86.9%	10.0%	10
1.1%	2.291902	2.294312	9.4	87.2%	10.3%	10
1.2%	2.391078	2.367117	9.5	88.7%	10.9%	10
1.3%	2.478204	2.471386	9.9	89.8%	9.7%	10
1.4%	2.497097	2.459727	9.7	90.1%	10.3%	15
1.5%	2.538775	2.539404	9.1	90.5%	10.3%	50
1.6%	2.547601	2.567571	8.9	90.7%	10.7%	50
1.7%	2.568953	2.589749	9.2	90.9%	10.6%	50
1.8%	2.604591	2.604230	8.7	91.2%	10.6%	150
1.9%	2.654309	2.641084	8.7	91.8%	10.7%	150
2.0%	2.681981	2.671089	8.9	92.0%	10.8%	150
2.1%	2.673861	2.680948	8.5	92.0%	11.2%	150
2.2%	2.717441	2.723774	8.2	92.4%	11.2%	150
2.3%	2.78469	2.790031	9.1	93.0%	11.6%	50
2.4%	2.771649	2.778416	8.3	92.8%	11.5%	50
2.5%	2.815806	2.862887	8.7	93.2%	10.8%	15
2.6%	2.837614	2.854453	8.0	93.3%	11.9%	15
2.7%	2.885975	2.895646	8.7	93.8%	11.4%	15

Pink or Purple fill: Parameters that best met target objectives

TP: presumed mean % of time that exposed individuals take effective precautions to avoid disease.
r0: basic reproduction number
AR: Attack rate
CFR: Case fatality rate
maxIll: Maximum number of persons ill at any one time

Other model assumptions as described in main text.

Misinformation making a disease outbreak worse: Outcomes compared for influenza, monkeypox and norovirus, S3: Additional results for Norovirus

Impacts on outbreak parameters from varying % of circulating advice that is bad advice.

BA%	Mean r0	r0 5%tile	r0 95%tile	Duration (wks)	AR	Peak AR	# of iterations
10.0%	0.993619	0.9515	1.028246	3.9	21.1%	3.9%	5
20.00%	1.085581	1.027619	1.165515	5.0	28.7%	4.2%	5
31.00%	1.381731	1.329291	1.43051	7.5	54.7%	6.9%	5
32.00%	1.383598	1.24947	1.540855	8.1	53.4%	5.7%	5
33.00%	1.446117	1.381129	1.576564	8.5	58.7%	7.0%	5
34.00%	1.485713	1.350396	1.555341	7.7	61.2%	7.4%	30
35.00%	1.541828	1.419516	1.68059	8.0	64.3%	7.5%	30
36.00%	1.587118	1.479341	1.723975	8.0	66.5%	7.9%	30
37.00%	1.637817	1.525191	1.763491	9.0	69.2%	8.4%	30
38.00%	1.688556	1.515316	1.83599	9.2	71.1%	8.2%	55
39.00%	1.765725	1.616062	1.922147	9.3	74.1%	8.4%	100
40.00%	1.856519	1.714502	2.001258	9.2	77.2%	8.8%	100
41.00%	1.924167	1.716357	2.072976	9.2	79.2%	8.9%	100
42.00%	1.995908	1.828731	2.142194	9.2	81.2%	9.1%	100
43.00%	2.061419	1.904316	2.239825	9.4	82.8%	9.4%	55
44.00%	2.151153	2.039615	2.264968	9.3	84.6%	9.3%	30
45.00%	2.207205	2.157404	2.268625	10.3	85.9%	10.5%	5
46.00%	2.403756	2.327897	2.501688	9.2	89.0%	9.9%	5

Pink or Purple fill: Parameters that best met target objectives

%BA: tested %s of total circulating advice that is bad advice
r0: basic reproduction number
AR: Attack rate
CFR: Case fatality rate

Other model assumptions as described in main text.

Misinformation making a disease outbreak worse: Outcomes compared for influenza, monkeypox and norovirus, S3: Additional results for Norovirus

Impacts on outbreak parameters from immunising specified %s of population against believing or circulating bad advice

imm%	Mean r0	r0 5%tile	r0 95%tile	Duration (wks)	AR	Peak AR	# of iterations
5.0%	2.4028	2.2255	2.5765	8.8	88.9%	10.5%	20
7.5%	2.2540	2.1037	2.3462	9.1	86.6%	9.5%	20
10.0%	2.1821	2.0333	2.3354	9.7	85.3%	9.5%	20
12.5%	2.0749	1.9571	2.1977	8.9	83.1%	9.5%	80
15.0%	2.0347	1.8862	2.1949	9.5	82.1%	9.3%	120
17.5%	1.9289	1.7991	2.0677	9.2	79.5%	8.9%	120
20.0%	1.8939	1.7662	1.9850	9.3	78.5%	8.9%	120
22.5%	1.8180	1.6610	1.9371	9.9	76.0%	8.6%	120
25.0%	1.7675	1.6597	1.8837	9.5	74.4%	8.5%	80
30.0%	1.6581	1.5766	1.7506	9.3	70.2%	8.0%	20
35.0%	1.5674	1.4540	1.6411	8.9	65.9%	7.6%	20
40.0%	1.4875	1.3682	1.5951	8.1	61.4%	7.3%	20
45.0%	1.4226	1.3286	1.5185	8.6	57.4%	7.1%	20
50.0%	1.3562	1.2280	1.4631	8.3	52.6%	6.4%	20
60.0%	1.3056	1.1967	1.4109	6.8	48.7%	6.3%	20
70.0%	1.1997	1.1460	1.2867	6.2	40.0%	5.6%	20
80.0%	1.1549	1.0928	1.2111	5.6	35.7%	5.3%	20
90.0%	1.1149	1.0273	1.2199	5.0	31.6%	4.8%	120

Pink or Purple fill: Parameters that best met target objectives

%imm: %s of population ‘immunised’ so that they neither believe nor share bad advice
r0: basic reproduction number
AR: Attack rate
CFR: Case fatality rate

Other model assumptions as described in main text.
